# The Alcohol Dependence Scale and DSM‐5 alcohol use disorder: Severity ratings correspond insufficiently in older patients

**DOI:** 10.1002/mpr.1811

**Published:** 2019-12-05

**Authors:** Anna Mejldal, Kjeld Andersen, Randi Bilberg, Barbara Braun, Michael Bogenschutz, Gerhard Bühringer, Anette Søgaard Nielsen, Behrendt Silke

**Affiliations:** ^1^ Unit of Clinical Alcohol Research, Institute of Clinical Research University of Southern DenmarkOdense, Denmark; ^2^ OPEN, Odense Patient data Explorative Network Odense University Hospital Odense Denmark; ^3^ IFT, Institut für Therapieforschung Munich Germany; ^4^ BRIDGE, Brain Research, Inter‐Disciplinary Guided Excellence, Department of Clinical Research University of Southern Denmark Odense Denmark; ^5^ Institute of Clinical Psychology and PsychotherapyTechnische Universität Dresden Dresden Germany; ^6^ Psychiatric Department Region of Southern Denmark Odense Denmark; ^7^ Bellevue Hospital Center New York USA

**Keywords:** alcohol dependence, assessment, diagnosis, older adults, severity

## Abstract

**Objectives:**

To investigate the psychometric properties of the frequently used Alcohol Dependence Scale (ADS) in older adults and the associations between ADS scores and alcohol use and DSM‐5 AUD symptom counts.

**Methods:**

Using baseline data from an international multicenter RCT on outpatient AUD treatment for adults aged 60+ with DSM‐5 alcohol use disorder (AUD; *n* = 529), we computed Cronbach's alpha (α) and applied confirmatory (CFA) and exploratory factor analysis (EFA) to determine the underlying factor structure. A structural equation model (SEM) explored the interrelationship of latent ADS factors with alcohol use and number of DSM‐5 criteria endorsed.

**Results:**

Internal consistency of the ADS (α = 0.81) was good. EFA revealed a three‐factor structure. Factor 1 (“Severe withdrawal symptoms”) consisted of severe psychoperceptual and psychophysical consequences of excessive drinking, Factor 2 (“Loss of control”) consisted of acute physical reactions of intoxication, and Factor 3 (“Obsessive–compulsive drinking”) described habitual drinking. The SEM suggested that only Factor 3 had large effects on DSM‐5 symptom score and drinking behavior.

**Conclusion:**

Lowering the ADS threshold or focusing on ADS items from Factor 3 may be more suitable measures of severity of alcohol dependence in treatment‐seeking older adults as ADS scores are low and not closely related to DSM‐5 AUD.

## 1 INTRODUCTION

The demographic change with aging populations, well underway in western‐industrialized countries, implies important public health challenges concerning the care for mental disorders in older adults, including treatment of alcohol use disorders (AUD). The prevalence rates for AUD and problematic alcohol consumption (e.g., binging) have increased among older adults (Bjørk, Vinther‐Larsen, Thygesen, Johansen, & Grønbæk, 2006; Han, Moore, Sherman, Keyes, & Palamar, 2017; Piontek, Gomes de Matos, Atzendorf, & Kraus, 2017). Moreover, substantial rates of problematic alcohol use and indication of AUD, by means of screening questionnaires among older adults, have been found in a variety of epidemiological studies from different western countries (Behrendt et al., 2017; K.‐L. Chou, Liang, & Mackenzie, 2011; K. L. Chou, Mackenzie, Liang, & Sareen, 2011; Geels et al., 2013; Grant et al., 2015; Han et al., 2017; Hapke, v der Lippe, & Gaertner, 2013; Piontek, Gomes de Matos, Atzendorf, & Kraus, 2016).

Diagnostic assessment is an essential part of care and treatment. Problems in diagnosing AUD in older adults could contribute to the reported lack of care and treatment (Grant et al., 2015; Mack et al., 2014) through sub‐optimal detection (Berner et al., 2007). With a well‐established diagnostic instrument, not specifically adapted to older age, paradoxically, the lowest lifetime prevalence rates for AUD are found in the age group with the longest exposure to alcohol availability—older adults (Grant et al., 2015). Markedly higher prevalence rates of AUD are found in a study with a diagnostic interview adapted to older adults compared with a study without such adaptation (Jacobi et al., 2015; Munoz et al., 2018). This indicates that even well‐established instruments may function suboptimally when applied in older adults without age‐specific adaptation.

Further, not only correct diagnosis is essential, but also correct assessment of the severity of AUD is important when planning treatment. The Alcohol Dependence Scale (ADS) has been developed to assess severity of the alcohol dependence syndrome as described by Edwards (Edwards, 1986; Ross, Gavin, & Skinner, 1990; H.A. Skinner & Horn, 1984). It includes the subscales “loss of control over consumption,” “compulsive drinking,” and “withdrawal” in adult alcohol‐dependent patients. These subscales are summed to one general ADS score, and overall, the ADS has very good internal consistency (Allen et al., 1994; Doyle & Donovan, 2009; Kivlahan, Sher, & Donovan, 1989). The raw score interpretation provides cutoff points of 1–13 suggesting a low level, 14–21 an intermediate level, 22–30 a substantial level, and 31–47 a severe level of alcohol dependence, respectively. A score of 9 or more is considered an indicator of a current DSM‐III diagnosis of alcohol abuse or dependence. However, factor structures vary across samples with different AUD diagnostic status and psychosocial characteristics (Kahler, Strong, Hayaki, Ramsey, & Brown, 2003; Kahler, Strong, Stuart, Moore, & Ramsey, 2003; Murphy & MacKillop, 2011). Importantly, relatively little is known to date on the psychometric properties of the ADS in older adults.

In early validation studies correlation with other established dimensional AUD measures, DSM‐III AUD and DSM‐III AUD symptom counts has been moderate to very good (Donovan, Kivlahan, Doyle, Longabaugh, & Greenfield, 2006; Doyle & Donovan, 2009; Ross et al., 1990). Correlation with AU patterns was more limited in some studies (Kivlahan et al., 1989; Ross et al., 1990). A study with older male veterans (Willenbring & Bielinski, 1994) found incongruences between DSM‐III alcohol dependence diagnosis and ADS scores. After the change to DSM‐5, the functioning of the ADS in older adults, in indicating AUD severity according to DSM‐5, to the best of our knowledge remains to be investigated.

In a recent RCT on older adults, ADS scores were surprisingly low given that all participants had DSM‐5 AUD (Andersen et al., in prep.; Behrendt et al., 2018). With the growing population of older adults and increasing prevalence of AUD among older adults, it is crucial to establish the feasibility of severity measures of AUD in this group.

Given this, we did an exploratory investigation of the reliability, validity, and factor structure of the ADS in a large clinical sample of older adults with DSM‐5 AUD. In addition, we investigated the latent factors of the ADS, and their interrelation with DSM‐5 AUD criteria count, and alcohol use in a data‐driven structural equation model (SEM) approach.

## 2 METHODS

### 2.1 Study design

This study utilized baseline data from the Elderly study; a multicenter, international, single‐blinded RCT detailed information has been provided elsewhere (Andersen et al., 2015). Elderly aimed to investigate the effects of two brief outpatient AUD interventions in adults aged 60+ years with DSM‐5 AUD. The study was conducted from 03/2014 to 03/2017 by four research centers (Odense, Denmark; Albuquerque, United States; and Munich and Dresden, Germany) and implemented at six study sites (Denmark: Copenhagen, Odense, and Aarhus; United States: Albuquerque; Germany: Munich and Dresden). DSM‐IV alcohol dependence was applied as inclusion criterion at the US site, and DSM‐5 AUD was applied in Denmark and Germany. To enable analyses with DSM‐5 AUD symptoms and symptom scores, the US subsample was excluded from the present analysis.

Data collection and management were conducted with the secure, web‐based “Research Electronic Data Capture” application for research data capture (Harris et al., 2009) (hosted at Odense University Hospital and the University of Southern Denmark in Odense).

Inclusion criteria were as follows: (a) age ≥ 60 years, (b) DSM‐5 AUD, and (c) ability to understand the study procedures. Exclusion criteria were as follows: (a) *current* psychotic symptoms, (b) *acute severe* major depression, (c) lifetime bipolar disorder, (d) *current* suicidal thoughts/behavior, (e) the use of illegal opioids and/or stimulants, (f) past 30 days psychosocial alcohol treatment (pure medical detoxification allowed), and (g) having a legally authorized representative.

In Denmark, individuals who contacted the alcohol treatment clinics were invited to participate. In Germany, participants were invited via media, general practitioners, hospitals, nursing homes, care services for the elderly, and congregations and by invitation flyers placed at libraries and community offices. Written informed consent was provided by participants before the baseline assessment.

ELDERLY was approved by the ethics committees at all involved research centers and registered in the http://ClinicalTrials.gov database (NCT02084173) (National Institutes of Health, 2016).

### 2.2 Assessment of in‐ and exclusion criteria

Mental disorders, suicidality, and psychotic symptoms were assessed with the Mini International Neuropsychiatric Interview (MINI) version 5.0.0 (Lecrubier et al., 1997). The Form 90 was used to assess illegal drug use and past 30 days AUD treatment (Miller & Del Boca, 1994). A tailor‐made “study quiz” was applied to assess capability of understanding the study procedures (at least eight correct answers out of 10), and tailor‐made questions were used to assess age and having a legally authorized representative.

### 2.3 Study sample

The baseline sample (*n* = 544) included *n* = 341 (62.7%) from Denmark and *n* = 203 (37.3%) from Germany. A total of 15 participants had incomplete ADS questionnaires, probably due to registration mistakes and therefore missing completely at random (MCAR). They were excluded from the analysis. The final sample consisted of *n* = 529 (331 from Denmark, 198 from Germany). At baseline, *n* = 64/529 had at least one missing value on DSM‐5 AUD criteria. This was mainly due to an interviewer error in Denmark, therefore missing at random (MAR). These missing values were handled by means of statistical imputation (details on this given in Section 2.6 Statistical Analysis).

### 2.4 Diagnostic assessment

Baseline assessments were conducted face to face at research or treatment sites or participants' homes. Interviewers (graduates or bachelor‐ or master‐level students in psychology) were trained and supervised in applying the assessment battery.

### 2.5 Measures

#### 2.5.1 ADS

Dimensional information on AUD severity was obtained with the ADS (H.A. Skinner & Horn, 1984). The ADS is a 25‐item questionnaire, aiming to provide a quantitative measure of alcohol dependence severity in the past 12 months. Scores range from 0 to 47, where 0 suggests no evidence of alcohol dependence. Scores of 1–13 reflect a low level of alcohol dependence, scores of 14–21 moderate levels, scores of 22–30 substantial levels, and scores of 31–47 severe alcohol dependence (H.A. Skinner & Horn, 1984).

#### 2.5.2 Form 90

Information on alcohol use (AU) patterns was obtained with Form 90 (Miller & Del Boca, 1994). Drinking days and drinks per drinking day were calculated over a 30‐day period by using day 60–31 prior to baseline assessment (referred to as “past 30 days AU”). Beforehand, this time span was considered to provide a more regular and, therefore, more accurate picture of pretreatment drinking pattern than the period leading up to baseline assessment where patients might reduce their drinking to be prepared for treatment. Past 30 days AU patterns were operationalized as number of drinking days, and the mean number of standard drinks a 12 g ethanol per drinking day (i.e., the sum of standard drinks over all days with alcohol use/number of days with alcohol use).

#### 2.5.3 DSM‐5 alcohol use disorder

Twelve months DSM‐5 AUD were assessed with the MINI. Two questions on *craving* were added to the MINI alcohol section for adaptation to DSM‐5 AUD criteria (American Psychiatric Association, 2013): “Did you have a craving for alcohol or an urge to drink that was so strong that you could not think of anything else?” and “Did you have a craving for alcohol or an urge to drink that was so strong that you could not resist it?”. DSM‐5 AUD severity was calculated as total number of DSM‐5 AUD criteria endorsed, the two craving questions counting as one, if any one of the questions was answered positively.

### 2.6 Statistical analysis

Cronbach's alpha was computed for the 25 ADS items to determine internal consistency. A value> = 0.7 was considered satisfactory.

Initially, we conducted confirmatory factor analysis (CFA) to test whether a one‐factor model would be a reasonable fit to the data. The CFA was based on the weighted least squares means and variance adjusted (WLSMV) estimation method to account for the binary and categorical ordinal nature of the items. The factor structure was evaluated based on goodness of fit measured by the χ^2^ statistic, the comparative fit index (CFI), the Tucker‐Lewis index (TLI), and the root mean square error of approximation (RMSEA). Although a nonsignificant χ^2^ is the aim, a significant χ^2^ can be expected due to the large sample size of the current study (Bentler & Bonett, 1980). For the RMSEA, lower values indicate a better fit, and values <0.06 are recommended as indicating a good fit. Values >0.95 for the CFI and > 0.95 for the TLI indicate a reasonable fit (Hooper, Coughlan, & Mullen, 2007; Kline, 2018; Yu, 2002).

After the CFA, we conducted exploratory factor analysis (EFA) to determine which number of factors would fit the data optimally. As the ADS consists of only binary and ordinal items, we based the EFA on the polychoric covariance matrix of the items. The Kaiser criterion (eigenvalues >1), the scree plot, and the Velicer MAP criterion were utilized to determine the appropriate number of factors to extract. After extracting the factors, we applied an oblique Promax rotation to allow for correlations between factors (Cattell, 1966; Fabrigar, Wegener, MacCallum, & Strahan, 1999; Zwick & Velicer, 1982).

The factors identified in the EFA were, thereafter, selected for SEM, where we added a structural model to explore the relationship between the found factors in the EFA, number of DSM‐5 AUD symptoms, drinking days, and drinks per drinking day. In several cases, single criteria of the 11 DSM‐5 AUD criteria were missing. These were assumed to be MAR and were addressed by the multivariate imputation by chained equations (MICE) method of multiple multivariate imputation. We imputed missing items using logistic regression. Age, sex, and country were included as auxiliary variables in the imputation model. A total of 20 imputed datasets were generated and analyzed separately, and point‐ and SE estimates were combined using the rules by Rubin (Rubin, 1987). To our knowledge, there are no implemented methods for combining SEM diagnostic criteria (χ^2^, RMSEA, CFI, and TLI) from multiple imputation. A pragmatic approach was, in this case, to evaluate model fit by examining the diagnostic criteria of the same SEM model from the complete‐case dataset and from all 20 imputed datasets.

All analysis was conducted using the Stata Software package 15.0 (StataCorp., 2015) and R (R Core Team, 2018). The EFA was conducted using the package psych (Revelle, 2018), CFA and SEM were conducted using the package lavaan (Rosseel, 2012), and MICE was conducted using the package mice (van Buuren & Groothuis‐Oudshoorn, 2011) in R.

## 3 RESULTS

### 3.1 Characteristics of the sample

The descriptive statistics of the 529 participants are provided in Table 1. The age of the participants ranged between 60 and 81 years, and 39.9% were women. The sample generally scored low on the ADS scale with a mean (standard deviation, SD) of 9.9 (6.0) with only 24.9% reaching a score of 14 or above and less than 5% reaching a substantial level of ADS alcohol dependence. On the other hand, the majority had severe DSM5 AUD (54.6%); that is, they endorsed six or more of the 11 DSM‐5 AUD criteria. The quartiles of the ADS were 6, 9, and 13.

**Table 1:** Baseline characteristics

Variable	
N	529
Age, mean (SD)	65.7 (4.4)
Sex [female, N (%)]	211 (39.9%)
Country, N (%)	
Danish Site	331 (62.6%)
German Site	198 (37.4%)
Number of drinking days over 30 days, mean (SD)†	21.6 (10.3)
Drinks per drinking day, mean (SD)†	8.7 (6.2)
ADS total score	
0	7 (1.3%)
1–13	390 (73.7%)
14–21	108 (20.4%)
22–30	23 (4.3%)
31–47	1 (0.2%)
ADS total score, mean (SD)	9.9 (6.0)
DSM5 AUD, total score‡	
Mild	81 (17.4%)
Moderate	130 (28.0%)
Severe	254 (54.6%)
DSM5 AUD, mean (SD) ‡	5.9 (2.8)
DSM5 AUD observed range ‡	2–11
†60–30 days prior to treatment, ‡64 missing due to interview error	

Response patterns on all 25 ADS items can be seen in Table 2. Only four of the 529 participants gave a positive answer on ADS item 21 “*As a result of drinking have you “felt things“ crawling on you that were not really there?”*, and only one of the four had experienced this several times. Therefore, it was necessary to exclude this item from all analysis, as it generated unstable solutions and problematic covariance matrices.

**Table 2:** ADS item endorsement

Item	Answer †	N(%)
1. How much did you drink the last time you drank?	Enough to get drunk	120 (22.7)
	Enough to pass out	16 (3.0)
2. Do you often have hangovers on Sunday or Monday mornings?	Yes	57 (10.8)
3. Have you had the shakes when sobering up (hands shaking and trembling inside)	Sometimes	157 (29.7)
	Often	92 (17.4)
4. Do you get physically sick (e.g., vomit and stomach cramps) as a result of drinking	Sometimes	100 (18.9)
	Often	25 (4.7)
5. Have you had the DTs (delirium tremens)—that is, seen, felt, or heard things	Sometimes	22 (4.2)
	Several times	6 (1.1)
6. When you drink, do you stumble about, stagger, and weave?	Sometimes	227 (42.9)
	Often	44 (8.3)
7. As a result of drinking, have you felt overly hot and sweaty (feverish)?	Once	50 (9.5)
	Several times	96 (18.1)
8. As a result of drinking, have you seen things that were not really there?	Once	13 (2.5)
	Several times	6 (1.1)
9. Do you panic because you fear you may not have a drink when you need it?	Yes	100 (18.9)
10. Have you had blackouts (loss of memory without passing out) as a result of drinking?	Sometimes	210 (39.7)
	Often	16 (3.0)
	Almost every time I drink	5 (0.9)
11. Do you carry a bottle with you or keep one close at hand?	Some of the time	76 (14.4)
	Most of the time	69 (13.0)
12. After a period of abstinence (not drinking), do you end up drinking heavily?	Sometimes	148 (28.0)
	Almost every time I drink	149 (28.2)
13. In the past 6 months, have you passed out as a result of drinking?	Once	66 (12.5)
	More than once	61 (11.5)
14. Have you had convulsions (fits) following a period of drinking?	Yes	11 (2.1)
	Several times	6 (1.1)
15. Do you drink throughout the day?	Yes	380 (71.8)
16. After drinking heavily, has your thinking been fuzzy or unclear?	Yes, but only for a few hours	190 (35.9)
	Yes, for one or 2 days	49 (9.3)
	Yes, for many days	15 (2.8)
17. As a result of drinking, have you felt your heart beating rapidly?	Yes	109 (20.6)
	Several times	47 (8.9)
18. Do you almost constantly think about drinking and alcohol?	Yes	128 (24.2)
19. As a result of drinking, have you heard things that were not really there?	Yes	7 (1.3)
	Several times	4 (0.8)
20. Have you had weird and frightening sensations when drinking?	Once or twice	19 (3.6)
	Often	2 (0.4)
21. As a result of drinking, have you felt things crawling on you that were not really there?	Yes	3 (0.6)
	Several times	1 (0.2)
22. With respect to blackouts (loss of memory): Has had blackouts that last	Less than an hour	137 (25.9)
	For several hours	75 (14.2)
	A day or more	9 (1.7)
23. Have you tried to cut down on your drinking and failed?	Once	65 (12.3)
	Several times	358 (67.7)
24. Do you gulp drinks (drink quickly)?	Yes	193 (36.5)
25. After taking one or two drinks, can you usually stop?	No	337 (63.7)

† Least severe answer possibility not displayed.

### 3.2 Cronbachs alpha and CFA

The internal consistency reliability estimate for the total ADS using standardized items was relatively high at 0.81. CFA did not clearly identify a one‐dimensional factor structure based on the χ^2^ (*p* < 0.0001), RMSEA (0.072, p (RMSEA<0.005) <0.0001), CFI (0.936), and TFL (0.930).

### 3.3 EFA

The EFA yielded seven eigenvalues greater than one. Based on the MAP criterion and examination of the scree plot and of the specific items and factor loadings, we judged that a three‐factor solution was the best fit to the data and the most interpretable. The cumulative variance, explained by the three factors, was 0.39. Factor loadings are reported in Table 3. All items but two had salient loadings (>0.30), the two being item 17 “*As a result of drinking, have you felt your heart beating rapidly?*” and item 2 *“Do you often have hangovers on Sunday or Monday mornings?”.* Three items had salient cross‐loadings on two factors, namely, item 1 “*How much did you drink the last time you drank?*”, which loaded on the first and second factor (0.32 and 0.45, respectively), and item 12 “*After a period of abstinence, do you end up drinking heavily?*”, which loaded on the second and third factor (0.41 and 0.34, respectively). Finally, item 3 “*Have you had the shakes when sobering up*?” loaded on factor 1 and factor 3 (0.31 and 0.43, respectively).

**Table 3:** Results of the EFA

	Alcohol Dependence Scale ‐ Items (Ordering from lowest number to highest within factor)	Factor loadings			Endorsement of factor in sample	
		Factor 1	Factor 2	Factor 3	mean (SD)†	observed range (possible range)
**Factor 1: Severe withdrawal symptoms**	19. As a result of drinking, have you heard “things” that were not really there?	**0.84**	−0.06	−0.12	0.89 (1.39)	0–14 (0–14)
	8. As a result of drinking, have you seen things that were not really there?	**0.82**	−0.03	−0.26		
	5. Have you had the delirium tremens (DTs), that is, seen, felt, or heard things?	**0.72**	−0.03	0.08		
	20. Have you had weird and frightening sensations when drinking?	**0.68**	0.14	−0.07		
	14. Have you had a convulsions (fits) following a period of drinking?	**0.50**	0.15	0.04		
	4. Do you get physically sick (e.g., vomit and stomach cramps) as a result of drinking?	**0.50**	0.03	0.22		
	17. As a result of drinking, have you felt your heart beating rapidly?	**0.23**	0.05	0.18		
**Factor 2: Loss of control**	10. Have you had blackouts (loss of memory without passing out) as a result of drinking?	0.11	**0.81**	−0.22	4.90 (3.74)	0–19 (0–20)
	13. In the past 6 months, have you passed out as a result of drinking?	0.06	**0.78**	−0.09		
	22. With respect to blackouts (loss of memory)	0.14	**0.76**	−0.25		
	16. After drinking heavily, has your thinking been fuzzy or unclear?	0.26	**0.50**	−0.02		
	25. After taking one or two drinks, can you usually stop?	−0.25	**0.50**	0.29		
	1. How much did you drink the last time you drank?	0.32	**0.45**	0.02		
	6. When you drink, do you stumble about, stagger, and weave?	0.18	**0.42**	0.11		
	12. After a period of abstinence (not drinking), do you end up drinking heavily?	−0.09	**0.41**	0.34		
	24. Do you gulp drinks (drink quickly)?	−0.11	**0.30**	0.17		
	2. Do you often have hangovers on Sunday or Monday mornings?	0.12	**0.24**	0.23		
**Factor 3: Obsessive‐compulsive drinking**	18. Do you almost constantly think about drinking and alcohol?	0.02	−0.10	**0.65**	4.13 (2.36)	0–11 (0–11)
	11. Do you carry a bottle with you or keep one close at hand?	−0.08	−0.08	**0.63**		
	15. Do you drink throughout the day?	0.13	−0.21	**0.60**		
	9. Do you panic because you fear you may not have a drink when you need it?	−0.02	0.12	**0.44**		
	23. Have you tried to cut down on your drinking and failed?	−0.15	0.03	**0.43**		
	3. Have you had the shakes when sobering up (hands trembling, shaking inside)?	0.31	0.12	**0.43**		
	7. As a result of drinking, have you felt overly hot and sweaty (feverish)?	0.21	−0.05	**0.34**		

† Mean score of items in factor

Factor 1 (“Severe withdrawal”), counting seven items, reflected very severe withdrawal symptoms, including hallucinations (items 19 and 8) and delirium tremens (item 5).

The second factor (“Loss of control”) included 10 items concerning the loss of control over drinking and experiences of blackouts (item 10) and passing out (item 13).

Lastly, Factor 3 (“Obsessive‐compulsive drinking”) contained seven items and reflected obsessive‐compulsive drinking habits such as constantly thing about alcohol (item 18) and drinking throughout the day (item 11).

We calculated the mean score for every factor separately as can be seen in Table 3. Endorsement of all items in Factor 1 “Severe withdrawal” was very low, on average participants endorsed less than one item. Mean scores for the two remaining factors were 4.90 and 4.13 for Factors 2 and 3, respectively.

### 3.4 SEM

A number of DSM‐5 symptoms, drinking days, and drinks per drinking day were entered as observed variables in the SEM with the above three factors as latent factors. Examination of the modification indices signified the need for covariance parameters between ADS item 10 “*Have you had blackouts (loss of memory without passing out) as a result of drinking*?” and item 22 “*With respect to blackout (loss of memory).*” In this exploratory analysis, we deemed the modification to make theoretical sense for the model as these two items refer to the same concept. This modification improved the model significantly using the χ^2^ difference test (χ^2^‐diff = 55.8, *p* < 0.0001).

**Figure 1:**
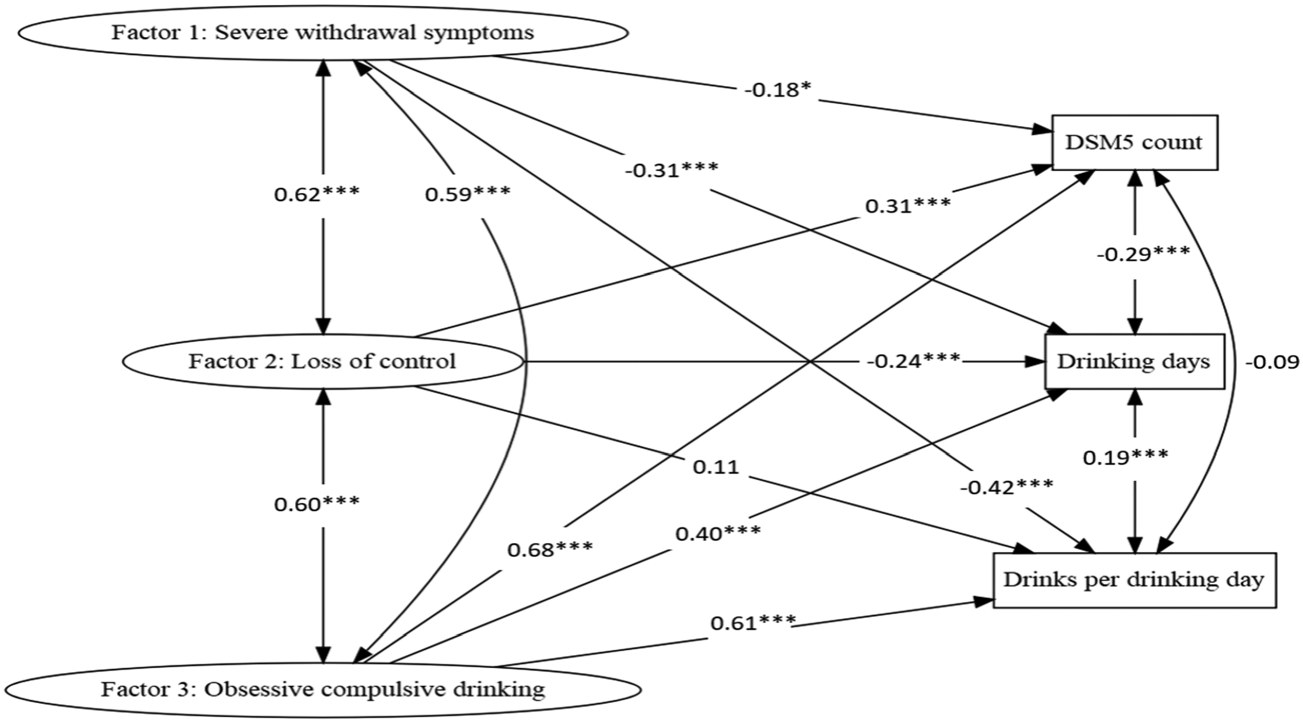
SEM structural partLegend: Numbers at single‐sided arrows indicate regression coefficients, and double sided arrows indicate correlations. *,** and *** indicate p‐value below 0.05, 0.01, and 0.001, respectively

When examining the fit indices from all 20 imputed datasets and the complete case, we found that they only differed on the third decimal and cutoff values for good fit were met in all cases, except for the χ^2^ as expected. For the complete case model, we found χ^2^ (p < 0.0001), RMSEA [0.070, p (RMSEA<0.005) = 0.45], CFI (0.968), and TFL (0.964). Correlations and regression coefficients can be seen in Figure 1. Standardized estimates and fit indices from the imputed datasets will be provided upon request.

In the SEM model, the three ADS factors were significantly, positively, and strongly correlated with each other, whereas the correlations between DSM‐5 symptom count, drinking days, and drinks per drinking day only had weak correlations; in the case of DSM‐5 count and drinking days, the correlation was even negative (−0.29, *p* < 0.001).

When investigating the standardized regression coefficients on DSM‐5 symptom count and drinking measures, results showed, when controlling for the first two factors, that the latent Factor 3 “Obsessive–compulsive drinking” had moderate to large effects on all three observed variables, especially on the number of DSM5 symptoms experienced (β = 0.68, p < 0.001) and drinks per drinking day (β = 0.61, *p* < 0.001). When controlling for Factors 2 and 3, Factor 1 “Severe withdrawal” displayed a weak negative effect on DSM5 symptom count (β = −0.18, *p* = 0.012) and moderate negative effects on drinking days (β = −0.31, *p* = 0.001) and drinks per drinking day (β = −0.42, p = 0.001). Finally, controlling for the other factors, latent Factor 2 “Loss of control” had no significant effect on drinks per drinking day and displayed a moderate effect on DSM5 symptom count (β = 0.31, p < 0.001) and a weak to moderate negative effect on drinking days (β = −0.24, *p* = 0.004).

## 4 DISCUSSION

This study evaluated the internal consistency and factor structure of the ADS in a large clinical sample of older adults with DSM‐5 AUD. In addition, we explored the interrelation between the latent factors of the ADS and DSM‐5 AUD symptom counts and alcohol use in a SEM.

In short, we have found that the ADS is moderately suitable to measure severity of alcohol dependence in older adults as the overall score is low and not overall closely related to DSM‐5 AUD and drinking measures. However internal consistency was very good, and we found three latent factors that measure a similar construct of the alcohol dependence score as in younger adults.

The internal consistency of the ADS in an old adult outpatient sample with DSM‐5 AUD is in‐line with internal consistency of the scales reported in studies with other age groups (Doyle & Donovan, 2009). An optimal fit factor wise was a three‐factor solution, “Severe withdrawal”, “Loss of control”, and “Obsessive‐compulsive drinking.” Number and content of factors support the factor structure found by Doyle et al. in the Combine and Match studies that have a younger sample (average age: 44.5 years) who met DSM‐IV criteria for alcohol dependence (Doyle & Donovan, 2009). Specifically, the factor “Loss of behavioral control and heavy drinking” in the study of Doyle et al. was entirely composed of the same ADS items as the factor “Loss of control” found in this study, and the factors “Obsessive‐compulsive drinking style” and “Psychoperceptual and psychophysical withdrawal” in Doyle et al. were, apart from 1 to 2 items, consistent with the factors “Obsessive–compulsive drinking” and “Severe withdrawal”, respectively. This indicates that, in older adults with DSM‐5 AUD, the ADS measures the same construct as in younger samples with AUD.

Of note, endorsement of ADS criteria in this older outpatient sample with classificatory 12‐month DSM‐5 AUD diagnosis was generally lower than in other studies with younger samples (Saxon, Kivlahan, Doyle, & Donovan, 2007) (Ross et al., 1990). This is similar to the low ADS scores and poor agreement of the ADS with DSM‐AUD diagnosis found by Willenbring et al. (Willenbring & Bielinski, 1994) in a relative old outpatient group of male veterans (average age: 66 years). The quartiles of the ADS (6, 9, and 13) in this study were substantially lower than the quartiles of 14, 22, and 31, which are recommended by the ADS interpretation guide by Skinner (H. A. Skinner, 1982). These were first reported in a study by Skinner and Allen in (Harvey A. Skinner & Allen, 1982) on 225 subjects (average age: 38 years) in outpatient treatment, though this study originally used a version of the ADS with 29 items and therefore has a higher attainable total score. The quartiles of 12, 17, and 22 of the regular 25‐item ADS found in the combine study (Saxon et al., 2007) (average age: 44.6 years) were somewhat lower than the original quartiles but still substantially higher than those in this study, with the upper quartile just barely reaching the lower quartile in the combine study.

The findings of Willenbring et al. and from our larger and more general sample of older adults may indicate that low ADS scores may be characteristic for older outpatient samples, possibly because of the double selection of well‐functioning individuals in outpatient treatment and the self‐selection bias of less chronically affected participants in old samples. However, this does not explain why ADS scores correspond poorly with DSM‐AUD diagnoses in our sample and in the study of Willenbring et al. While the latter study applied lifetime criteria that may have contributed to the limited correspondence, 12‐month DSM‐5 AUD criteria were applied in Elderly, yet the same lack of correspondence was found. This may in part be due to the integration of three former DSM alcohol abuse criteria in the DSM‐5 AUD diagnosis, as the ADS was not designed to cover the psychosocial aspects covered by these (Edwards, 1986; Hasin et al., 2013). The former abuse symptom “interpersonal conflict” was quite prevalent in the Elderly study (Behrendt et al., 2018).

In order to further investigate the possible reasons for the lack of correspondence between ADS scores and DSM‐5 AUD diagnoses, we explored the relationship between ADS factors and DSM‐5 AUD symptom counts and drinking patterns in a SEM model. Interestingly, results showed that

the factor “obsessive–compulsive drinking” was significantly and positively related to DSM‐5 symptom count and drinking measures, especially to drinks per drinking day. However, the two remaining factors had weaker and even negative effects. A possible explanation of the association between the factor “obsessive–compulsive drinking” and DSM‐5 AUD could be that the items in this factor have similarities to the items in the DSM‐5. For example, item 23 *“Have you tried to cut down on your drinking and failed?*” is identical to item 2 in DSM‐5 *“There is a persistent desire or unsuccessful efforts to cut down or control alcohol use”* (American Psychiatric & American Psychiatric Association, 2013).

The factor “Severe withdrawal” had a negative relation to drinking days and drinks per drinking day. This could be because patients generally endorsed very few items in this factor and those who did had high probabilities of endorsing the other two factors. One might speculate that patients experiencing severe withdrawal symptoms could be forced to take stretches of several days between drinking sprees to recover or to try and control their consumption, thus reporting a lower overall intake of alcohol. A study by Wood et al. (Wood, Sobell, Sobell, Dornheim, & Agrawal, 2003) supports this, finding that nondaily drinkers score higher on the ADS than daily drinkers, although their reported number of drinks per drinking day did not differ significantly. Also, the reverse relation between “severe withdrawal” and drinking measures could simply be due to patients regularly consuming alcohol, thereby not experiencing withdrawal symptoms.

It might be that the ADS captures a much more severe end of the spectrum of AUD compared with DSM‐5. As for DSM‐5 symptoms, the lack of an association could be because DSM‐criteria cover a broader range of behavioral and psychological symptoms that are not covered by Factor 1 “Severe withdrawal.” Also, Saxon et al. (Saxon et al., 2007) have pointed out that DSM‐IV physical dependence and withdrawal in the ADS may cover different constructs. Moreover, in our sample, roughly 20% reported age of AUD onset after the age of 60 years (Behrendt et al., 2018). Late onset is generally atypical (Behrendt, Wittchen, Höfler, Lieb, & Beesdo, 2008; Wagner & Anthony, 2002), though maybe not surprising in this sample. Some adults with early onset might be so impaired that outpatient treatment no longer is an option for them at this age. Others might already have succumbed to secondary diseases related to AUD—both possibly introducing a selection bias toward more well‐functioning individuals with later AUD onset. Thus, they may not yet have developed the severe withdrawal symptoms associated with a chronic course of AUD. This fact might also explain the low endorsement of items in the “Severe withdrawal” factor, as also noted in (Barry, Blow, & Oslin, 2002; A. Kuerbis, Sacco, Blazer, & Moore, 2014). The other two factors may depend less on the duration of AUD and more on the effects of heavy alcohol use in older age, as older adults often will experience alcohol‐induced effects at lower levels of intake because of increased sensitivity to alcohol with aging (Barry et al., 2002).

### 4.1 Limitations

This study had a number of limitations. First, we analyzed only treatment seeking, DSM‐5 AUD participants who met inclusion and exclusion criteria for the Elderly Study. However, exclusion criteria were few to increase external validity. The participants of the Elderly sample had relatively late age of onset and high educational status (Behrendt et al., 2018). Thus, there was some degree of self‐selection. Results are not generalizable to other regions, other patient groups, the general population, or very old or institutionalized seniors. Furthermore, the sample size was not sufficiently large to analyze possible differences between subgroups, such as differences across the two countries. We compared the ADS with DSM‐5 AUD criteria. These have disadvantages when used with older in comparison with younger adults, as differential item functioning (A. N. Kuerbis, Hagman, & Sacco, 2013). However, Kuerbis et al. showed that older adults need to have more, not less, severe AUD to endorse DSM‐5 AUD symptoms than younger adults. Therefore, the relatively high DSM‐5 symptom counts in our sample are likely not due to false positive DSM‐5 criterion endorsement.

Finally, the EFA and SEM are used in an explorative data‐driven fashion, and therefore, any conclusions drawn are tentative. Yet, results from the EFA were consistent with previous findings.

## 5 CONCLUSION

We have found that the ADS is moderately suitable to measure the severity of alcohol dependence in treatment‐seeking older adults as the overall score is low and not overall closely related to DSM‐5 AUD symptom counts and drinking measures. In older outpatients with DSM‐5 AUD, ADS items related to obsessive‐compulsive drinking are most in agreement with currently applied DSM‐5 AUD and drinking measures and may thus be singled out for diagnostic application as the most feasible indicator of DSM‐5 AUD severity in this population.

### Implications

Based on these results, a recommendation would be to lower ADS thresholds for levels of alcohol dependence severity based on the ADS for older adults. This may also lead to a better convergence with DSM‐5 AUD severity. In clinical assessments with older adults and especially in well‐functioning outpatients, a low overall ADS score should not be regarded as an indication of low AUD severity according to DSM‐5. In this age group, scoring on Factor “3” should always be paid clinical attention to in addition to the overall ASD score, because scorings on this factor may reveal substantial behavioral and psychological problems with drinking that demand intervention.

## FUNDING AND DECLARATION OF INTEREST

This study was funded by the Lundbeck Foundation, Denmark, which had no role in study design, collection, analysis and interpretation of data, writing of the report, or decision to submit the article for publication.

Gerhard Bühringer has no conflict of interest related to the study topic. However, he has received unrestricted gambling research grants from the Bavarian State Ministry of Finance (regulatory authority for and operator of the State Gambling Monopoly) via the Bavarian State Ministry of Public Health and Care Services, from the German Federal Ministry of Economics and Technology (regulatory authority for parts of the commercial gambling industry) and from public and commercial gambling providers.

Michael Bogenschutz has received research grants from the National Institute on Alcohol Abuse and Alcoholism, the National Institute on Drug Addiction, the Heffter Research Institute, and private individuals. The other authors report that they do not have a conflict of interest.
